# Coating-Dependent Effects of Silver Nanoparticles on Tobacco Seed Germination and Early Growth

**DOI:** 10.3390/ijms21103441

**Published:** 2020-05-13

**Authors:** Renata Biba, Dajana Matić, Daniel Mark Lyons, Petra Peharec Štefanić, Petra Cvjetko, Mirta Tkalec, Dubravko Pavoković, Ilse Letofsky-Papst, Biljana Balen

**Affiliations:** 1 Department of Biology, Faculty of Science, University of Zagreb, Horvatovac 102a, 10000 Zagreb, Croatia; renata.biba@biol.pmf.hr (R.B.); dana.matich@gmail.com (D.M.); ppeharec@biol.pmf.hr (P.P.Š.); pcvjetko@biol.pmf.hr (P.C.); mtkalec@biol.pmf.hr (M.T.); dubravko@biol.pmf.hr (D.P.); 2 Center for Marine Research, Ruđer Bošković Institute, G. Paliaga 5, 52210 Rovinj, Croatia; lyons@irb.hr; 3 Institute of Electron Microscopy and Nanoanalysis (FELMI), Graz University of Technology, Graz Centre for Electron Microscopy (ZFE), Austrian Cooperative Research (ACR), Steyrergasse 17, 8010 Graz, Austria; ilse.papst@felmi-zfe.at

**Keywords:** silver nanoparticles, AgNP stability, *Nicotiana tabacum* L., germination, root length, fresh and dry weight

## Abstract

Silver nanoparticles (AgNPs) are used in a wide range of consumer products because of their excellent antimicrobial properties. AgNPs released into the environment are prone to transformations such as aggregation, oxidation, or dissolution so they are often stabilised by coatings that affect their physico-chemical properties and change their effect on living organisms. In this study we investigated the stability of polyvinylpyrrolidone (PVP) and cetyltrimethylammonium bromide (CTAB) coated AgNPs in an exposure medium, as well as their effect on tobacco germination and early growth. AgNP-CTAB was found to be more stable in the solid Murashige and Skoog (MS) medium compared to AgNP-PVP. The uptake and accumulation of silver in seedlings was equally efficient after exposure to both types of AgNPs. However, AgNP-PVP induced only mild toxicity on seedlings growth, while AgNP-CTAB caused severe negative effects on all parameters, even compared to AgNO_3_. Moreover, CTAB coating itself exerted negative effects on growth. Cysteine addition generally alleviated AgNP-PVP-induced negative effects, while it failed to improve germination and growth parameters after exposure to AgNP-CTAB. These results suggest that the toxic effects of AgNP-PVP are mainly a consequence of release of Ag^+^ ions, while phytotoxicity of AgNP-CTAB can rather be ascribed to surface coating itself.

## 1. Introduction

Over the past two decades the application of nanomaterials (NMs) in a broad range of industries as well as in many aspects of daily life has gradually expanded because of their unique chemical and physical characteristics [1]. Among various types of nanomaterials, silver nanoparticles (AgNPs) are used in around 25% of NM-containing consumer products because of their excellent antibacterial and antifungal protection, with applications ranging from household items, food packaging, textiles, and medical equipment to agriculture and many types of electronic devices [2,3,4,5,6].

Release of AgNPs in the environment can occur in various stages of their cycle, from production and manufacturing to finished products and during their eventual disposal [7,8,9]. Levels of AgNPs in the environment are difficult to determine as they undergo many reactions that include dissolution, aggregation and chemical complexation which ultimately change their speciation and affect the bioavailability of silver [9,10,11]. As a further level of complexity, organic or inorganic compounds that are used for AgNPs stabilisation against aggregation greatly affect their physico-chemical properties [12,13] and can consequently change their behaviour in the environment. Through significant increase of AgNP content in soil, water, and air, their uptake and accumulation in plants is inevitable. In addition, increased application of nanomaterials in agriculture and development of nano pesticides represents a more direct path for plant contact with AgNPs [14,15]. Uptake, biotransformation, and translocation of AgNPs by plants could serve as a pathway for their transport and bioaccumulation in food chains [16], thus creating a risk for the whole ecosystem.

Many studies have already shown AgNP phytotoxic effects that have been mainly attributed to the excess production of reactive oxygen species (ROS), leading to increased oxidative damage to lipids, proteins, and DNA [1,17,18,19]. However, studies that investigated the impact of AgNPs on plant germination, growth, and morphology showed both stimulatory [20,21] and detrimental [22,23] effects. Such variance likely arises from a number of factors that determine AgNP toxicity, predominantly plant species, composition, and concentration of tested AgNPs, growth stage of plant and experimental setup, especially the composition of plant growing media [24]. Due to increased utilisation of AgNPs in agriculture, it is important to investigate their effects on seed germination and early growth of seedlings, the critical stages for the successful growth of plants.

In the present study we have investigated the stability of differently coated AgNPs in a nutrient medium, as well as their effect on germination and early growth of tobacco (*Nicotiana tabacum* L.), an important crop plant that is commonly used as a model organism in abiotic stress research [18,19,25,26]. Tobacco seeds were sown on a nutrient media supplemented with the same concentrations of laboratory-synthesised AgNPs stabilised with two types of surface coatings, polyvinylpyrrolidone (PVP; AgNP-PVP) and cetyltrimethylammonium bromide (CTAB; AgNP-CTAB). The effects of coating molecules (PVP and CTAB) alone were also examined. To determine possible differences in effects on germination and seedling growth between silver applied in the form of nanoparticles and its ionic form, seeds were also exposed to the same concentrations of AgNO_3_ as well as to combined treatments with cysteine, a ligand with a strong affinity for silver [27,28].

## 2. Results

### 2.1. Silver Nanoparticles (AgNP) Characterisation

**Figure 1:**
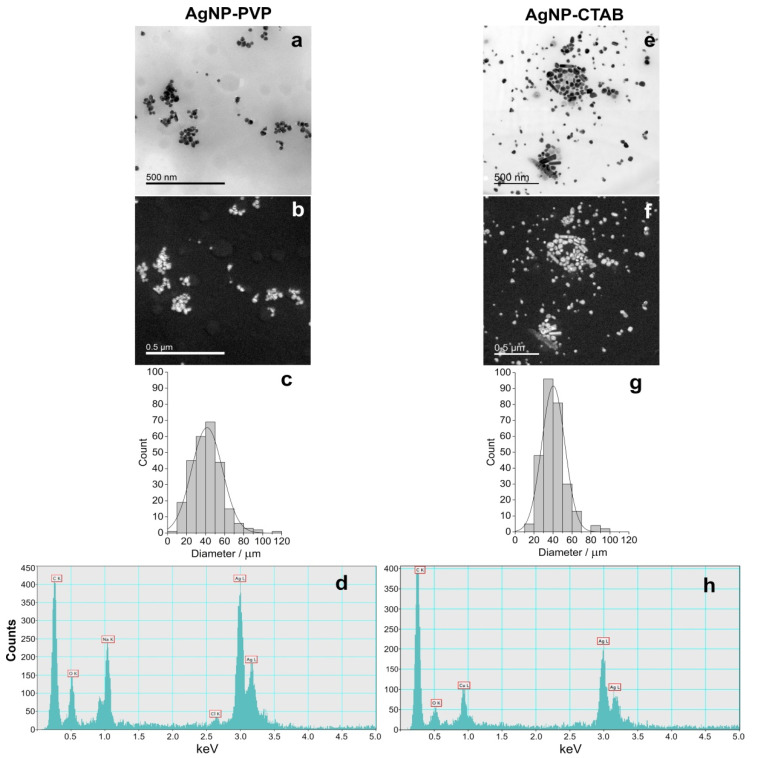
Stock solutions of silver nanoparticles (AgNPs) coated with either polyvinylpyrrolidone (PVP; AgNP-PVP) or cetyltrimethylammonium bromide (CTAB; AgNP-CTAB) in ultrapure water investigated by transmission electron microscopy (TEM); (**a**,**e**) bright field image, (**b**,**f**) silver elemental map, (**c**,**g**) size distribution histograms, (**d**,**h**) energy-dispersive X-ray spectrum. For each stock solution, 4 replicas (*n* = 4) were analysed.

Synthesised AgNP-PVP and AgNP-CTAB were characterised using UV-Vis spectroscopy, TEM, dynamic light scattering (DLS), and electrophoretic light scattering (ELS), and the results are presented in Figure 1 and Table 1. UV-Vis spectra showed surface plasmon resonance (SPR) peaks of AgNP-PVP and AgNP-CTAB at 420 and 434 nm, respectively, confirming the synthesis of nanosized silver particle dispersions. These SPR absorption peaks are consistent with silver nanoparticles of 50 and 60 nm diameters, respectively. Acquired TEM images revealed that both AgNP-PVP (Figure 1a) and AgNP-CTAB (Figure 1e) generally had a spherical shape with a small percentage of rod like particles. Silver elemental map (Figure 1b,f) and energy dispersive X-ray (EDX) analysis (Figure 1d,h) confirmed that all detected particles contained silver. Particle size histograms for AgNP-PVP and Ag-CTAB (Figure 1c,g) derived from transmission electron micrographs indicate mean (± S.D.) diameters of 41.12 ± 16.25 nm and 40.30 ± 12.16 nm, respectively. The mean (± S.D.) hydrodynamic diameters of the AgNP-PVP dispersion were 41.53 ± 1.14 nm (consistent with TEM and UV-Vis data) and 8.16 ± 0.37 nm. The latter size distribution likely represents PVP micelles in solution deriving from the PVP used in nanoparticle synthesis. The mean (± S.D.) size distribution of AgNP-CTAB was trimodal, with the primary size distribution of 64.75 ± 7.28 nm consistent with UV-Vis data. The peak noted at 26.28 ± 6.37 nm may be related to the presence of some smaller particles which may have formed and been stabilised at the very beginning of the synthesis reaction when silver reduction was occurring in the presence of a large excess of CTAB, while the peak at 227.99 ± 16.02 nm represents some small agglomerates present. The measured ζ potential value of AgNP-PVP was −1.0 ± 0.6 mV, while stabilisation with cetyltrimethylammonium cation led to a positive overall charge and ζ potential value of 11.4 ± 1.1 mV.

### 2.2. AgNP Stability in Exposure Medium

**Figure 2:**
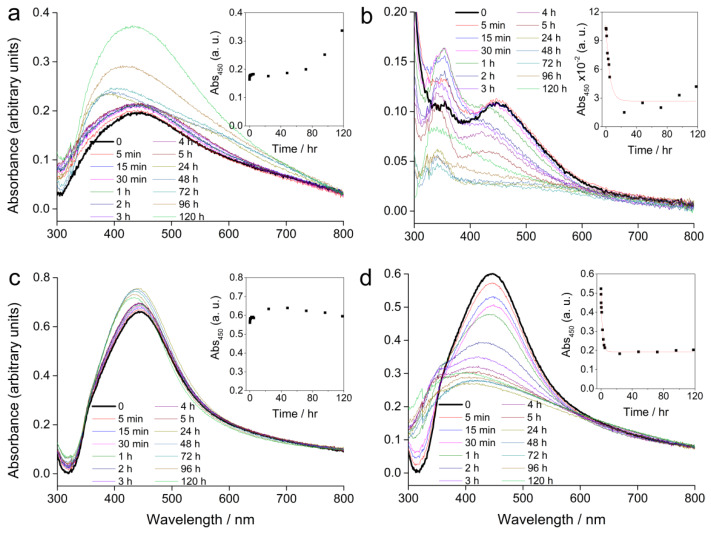
UV-Vis absorption spectra of AgNPs alone or in combination with cysteine in a solid ½ strength Murashige and Skoog (MS) medium recorded over a period of five days; (**a**) 100 µM AgNP-PVP, (**b**) 100 µM AgNP-PVP with 500 µM cysteine, (**c**) 100 µM AgNP-CTAB, and (**d**) 100 µM AgNP-CTAB with 500 µM cysteine. Insets show the temporal change in absorbance at 450 nm. (The step at about 320 nm is an instrument artefact and not due to species in solution).

The absorbance spectra of AgNP-PVP and AgNP-CTAB (100 µM), alone and in combination with 500 µM cysteine, in a solid ½ strength Murashige and Skoog (MS) medium are shown in Figure 2.

Compared to the SPR absorbance maximum of the stock solution, AgNP-PVP showed a peak shift from 420 to about 450 nm immediately after the solidification of the ½ strength MS medium (Figure 2a). Over several hours the position of the peak did not significantly change, but a slight increase in the absorbance value was recorded. Further measurements over several days showed a change in the peak position back towards lower wavelengths, while the absorbance continued to rise. This is consistent with some dissolution of the nanoparticles, as smaller sizes display a SPR at lower wavelengths with a more intense absorbance peak. Addition of cysteine to AgNP-PVP led to an exponential decrease of the SPR intensity over 24 h suggesting rapid agglomeration, while a shift to lower wavelengths indicated some dissolution of the AgNPs (Figure 2b). Concomitantly, the appearance of an additional peak at around 350 nm after 5 min, which continued to increase in intensity over several days, was noted. This peak may be related to formation of species such as silver clusters derived from Ag^+^ ions released from the AgNPs, or very small particles.

AgNP-CTAB (Figure 2c) showed better stability in a solid ½ strength MS medium compared to AgNP-PVP. Even though there was an immediate shift in the absorption maximum (434 to 442 nm) the intensity of the SPR peak remained relatively constant over 5 days suggesting little agglomeration had occurred. Measurement after 24 h showed that the absorbance maximum slightly shifted towards lower wavelengths with an accompanying slight increase in absorbance intensity. This is consistent with a small amount of dissolution of the AgNPs. Unlike the case for AgNP-CTAB alone, addition of cysteine was followed by a rapid decrease in SPR intensity (Figure 2d) indicating rapid agglomeration, while a shift towards lower wavelengths was consistent with reduction of particle size through release of Ag^+^ ions due to the effect of cysteine. The temporal trend in absorbance at 450 nm is shown for each sample in the insets in Figure 2. Addition of cysteine to AgNP-PVP and AgNP-CTAB resulted in a rapid decrease in SPR intensity that could be modelled by the function:

y = A.exp(−x/t) + y_0_, (1)

(residual R^2^ = 0.96 and 0.99, respectively), with rate constant k given by:

k = 1/t.(2)

The rate constants for AgNP-PVP and AgNP-CTAB were 0.21 and 0.52 hr^−1^, respectively, indicating that dissolution in media with cysteine occurs more rapidly for the CTAB-coated nanoparticles compared to those coated with PVP.

### 2.3. Germination Parameters

To examine the effects of AgNP-PVP, AgNP-CTAB, and AgNO_3_ alone and in combination with cysteine as well as of coatings (PVP and CTAB) on germination of tobacco seeds, three different parameters were evaluated: germination percentage, germination rate, and germination index. The germination percentage showed treatment effect (F = 13.14, *p* < 0.001), while germination rate and germination index were affected by both concentration (F = 8.25, *p* < 0.005 and F = 4.92, *p* < 0.05, respectively) and treatment (F = 70.04, *p* < 0.001 and F = 58.99, *p* < 0.001, respectively).

**Figure 3:**
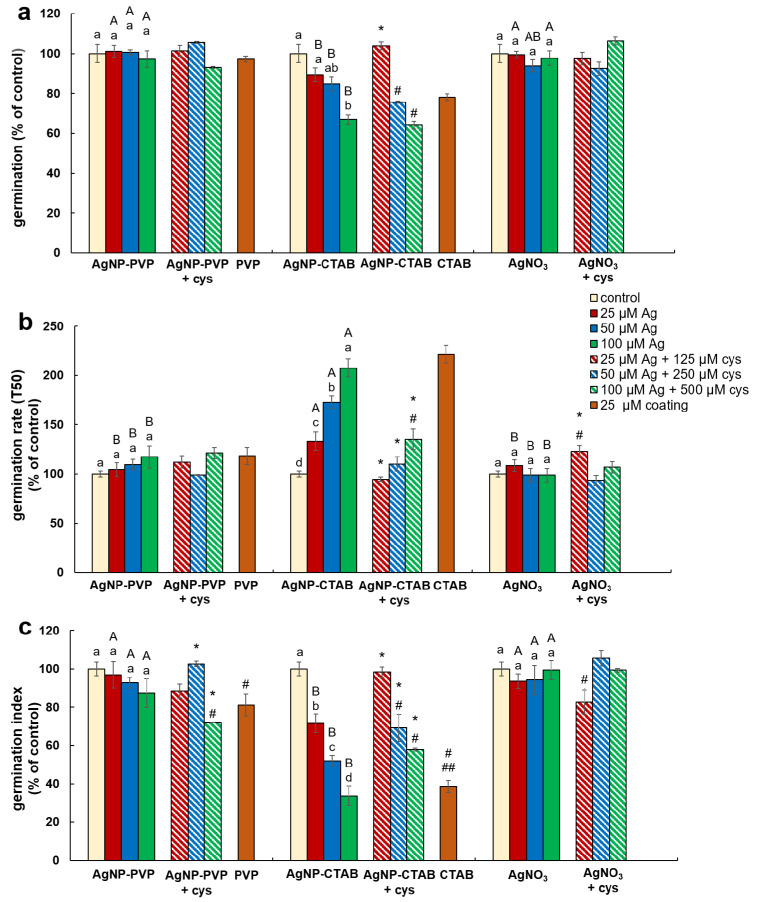
Germination parameters of tobacco seeds after 5 days of germination on a solid ½ strength MS medium supplemented with 25, 50 and 100 μM AgNP-PVP, AgNP-CTAB, and AgNO_3_ and their combination with 125, 250 and 500 μM cysteine (cys), as well as 25 μM polyvinylpyrrolidone (PVP) and cetyltrimethylammonium bromide (CTAB) coatings alone. (**a**) germination percentage, (**b**) germination rate (T_50_), and (**c**) germination index. Values are the means ± standard error of three different experiments with 100 seeds per experiment (*n* = 300). If columns are marked with different letters, the treatments are significantly different at *p* ≤ 0.05 (a two-way ANOVA followed by Duncan post-hoc test); small letters mark the differences among different concentrations of the same treatment type as well as control while capital letters mark the differences among different treatment types of the same concentration. Asterisk (*) denotes significant differences among treatments with and without cysteine of the corresponding concentration; hash sign (#) denotes significant difference between each treatment with cysteine and control; and double hash sign (##) denotes significant difference between the two coatings.

Compared to control, a significant decrease of germination percentage of tobacco seeds was noted only in the treatment with the highest concentration (100 µM) of AgNP-CTAB, where it decreased by 40% (Figure 3a). Comparison between the treatments of different forms of silver (AgNP-PVP, AgNP-CTAB or AgNO_3_) of the same concentration revealed that exposure to all AgNP-CTAB concentrations caused significantly stronger impact on seed germination compared to corresponding treatments with AgNP-PVP and almost all treatments with AgNO_3_ (Figure 3a). Exposure to 25 µM CTAB coating also significantly reduced germination percentage compared to control (Figure 3a). Furthermore, germination rate (Figure 3b) and germination index (Figure 3c) indicated that germination was significantly delayed and slower in all AgNP-CTAB treatments compared to the control as well as to the corresponding treatments with AgNP-PVP and AgNO_3_. The same effect was also noticed after exposure to 25 µM CTAB coating alone for both parameters compared to control (Figure 3b,c), although 25 µM PVP coating also reduced germination index, but not as severe as CTAB (Figure 3c). Addition of cysteine to AgNP-CTAB treatments significantly alleviated the AgNP-CTAB impact on germination percentage, but only at the lowest applied concentration (25 µM) (Figure 3a). Germination rate and germination index were significantly higher in all three combined treatments with cysteine compared to corresponding treatments with AgNP-CTAB alone, although germination was still significantly delayed compared to control on higher tested concentrations (Figure 3b,c).

### 2.4. Growth Parameters

Effects of AgNP-PVP, AgNP-CTAB, and AgNO_3_ alone and in combination with cysteine as well as of coatings (PVP and CTAB) on seedling growth were evaluated by root length reduction percentage, fresh mass reduction percentage, and dry matter content percentage.

**Figure 4:**
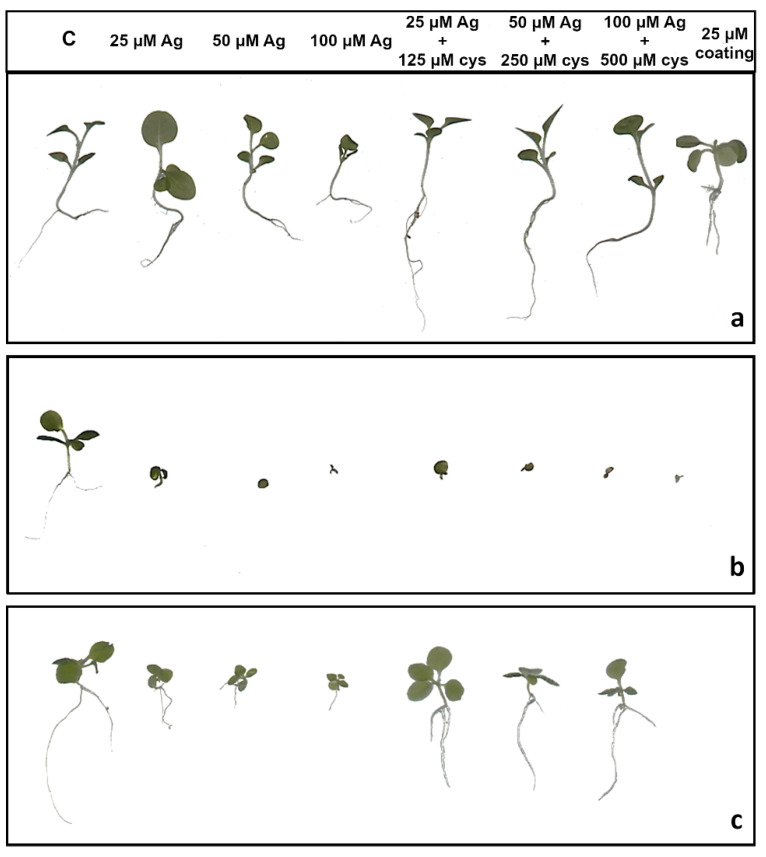
Three weeks old tobacco seedlings after growing on a solid ½ strength MS medium supplemented with 25, 50 and 100 μM AgNPs or AgNO_3_ and their combination with 125, 250, and 500 μM cysteine (cys), as well as 25 μM PVP and CTAB coatings alone. (**a**) AgNP-PVP, (**b**) AgNP-CTAB, and (**c**) AgNO_3_. C—control plant.

**Figure 5:**
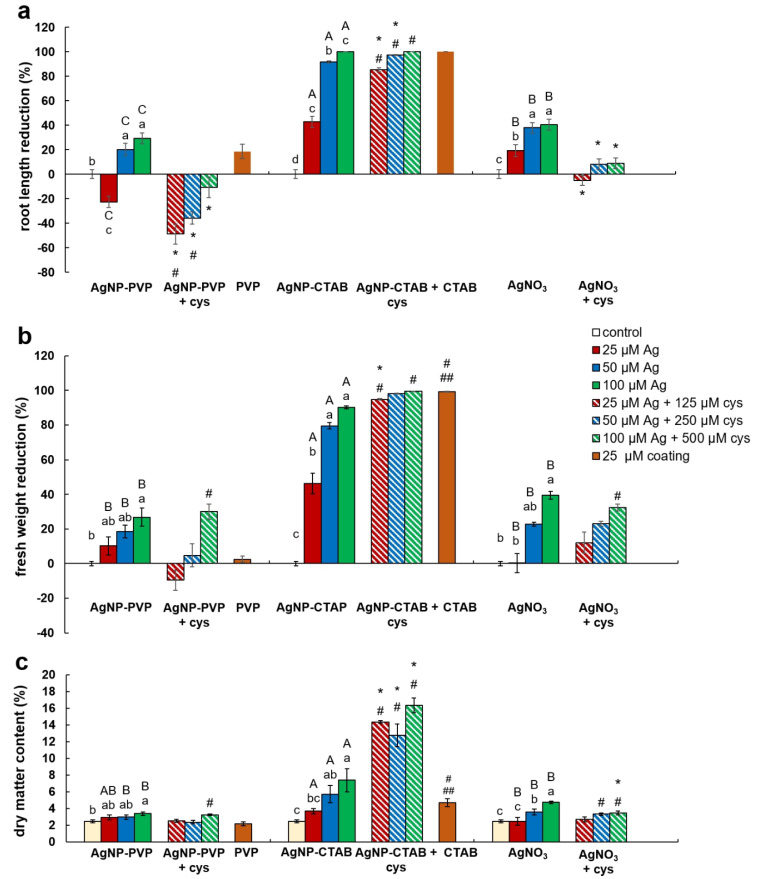
Growth parameters of three weeks old tobacco seedlings after growing on a solid ½ strength MS medium supplemented with 25, 50, and 100 μM AgNP-PVP, AgNP-CTAB, and AgNO_3_ and their combination with 125, 250, and 500 μM cysteine, as well as 25 μM PVP and CTAB coatings alone. (**a**) root length reduction percentage, (**b**) fresh weight reduction percentage, and (**c**) dry matter content. For root length, values are the means ± standard error of three different experiments with 20 seedlings per experiment (*n* = 60). For fresh and dry weight, values are the means ± SE of three different experiments with 3 replicas per experiment (*n* = 9). If columns are marked with different letters, the treatments are significantly different at *p* ≤ 0.05 (a two-way ANOVA followed by Duncan post-hoc test); small letters mark the differences among different concentrations of the same treatment type as well as control while capital letters mark the differences among different treatment types of the same concentration. Asterisk (*) denotes significant differences among treatments with and without cysteine of the corresponding concentration; hash sign (#) denotes significant difference between each treatment with cysteine and control; and double hash sign (##) denotes significant difference between the two coatings.

Root length reduction was affected by concentration (F = 98.62, *p* < 0.0001) as well as treatment (F = 225.09, *p* < 0.0001). Root length of tobacco seedlings decreased with the increased concentration of both types of AgNPs or AgNO_3_ (Figure 4, Figure 5a). Significant inhibition of root growth was present in the AgNP-CTAB and AgNO_3_ treatments from the lowest tested concentration compared to the control. However, all AgNP-CTAB concentrations significantly reduced root growth compared to corresponding treatments with AgNP-PVP and AgNO_3_ (Figure 4, Figure 5a). The weakest effect was obtained after exposure to AgNP-PVP; interestingly, treatment with 25 µM AgNP-PVP significantly enhanced root growth, while the 50 and 100 µM concentrations significantly reduced it (Figure 5a). Exposure to 25 µM PVP and CTAB coatings alone resulted in a significant decrease in root growth compared to the control, although the effects of CTAB were far more detrimental than those of PVP (Figure 4, Figure 5a). Addition of cysteine significantly reduced the negative effects of all AgNP-PVP and AgNO_3_ treatments; namely, after exposure to all AgNP-PVP concentrations as well as to two lower AgNO_3_ concentrations (25 and 50 µM) in combination with cysteine rescued root growth (root length similar to the control) was noticed. Moreover, in the case of 25 and 50 µM AgNP-PVP the root growth even improved compared to control (Figure 4, Figure 5a). On the contrary, combined treatments of AgNP-CTAB and cysteine failed to alleviate negative effects of AgNP-CTAB; compared to corresponding treatments with AgNP-CTAB alone, even lower values were recorded (Figure 5a).

Fresh weight reduction was affected by concentration (F = 12.23, *p* < 0.0001) as well as treatment (F = 40.02, *p* < 0.0001). Although the percentage of fresh mass of tobacco seedlings reduced with the increase of AgNP-PVP and AgNO_3_ concentrations, only the highest 100 µM concentration significantly reduced the fresh weight compared to the control (Figure 5b). Addition of cysteine to lower concentrations of AgNP-PVP (25 and 50 µM) somewhat alleviated the negative effect on fresh weight (Figure 5b, Figure S1), although not statistically significant compared to treatments without cysteine. Additionally, addition of cysteine did not improve the effect of AgNO_3_ treatments on fresh weight reduction. All treatments with AgNP-CTAB caused the highest reduction of fresh weight compared to other two treatments; ranging between 40% at the lowest (25 µM) and 90% at the highest (100 µM) concentration (Figure 5b, Figure S1). Cysteine addition failed to improve shoot growth; on the contrary, even higher fresh weight reduction was obtained after treatments of 25 and 50 µM AgNP-CTAB with cysteine than with AgNP-CTAB alone (Figure 5b). Exposure to 25 µM CTAB coating also resulted in a significant decrease in fresh mass, unlike PVP coating which had no effect on seedling growth (Figure 5b, Figure S1).

Dry matter content was affected by both, concentration (F = 8.41, *p* < 0.001) and treatment (F = 12.99, *p* < 0.001). It showed significant increase in seedlings exposed to AgNP-CTAB (Figure 5c). Those effects were not affected with cysteine; moreover, after exposure to 25 µM AgNP-CTAB and 125 µM cysteine dry matter content was even higher compared to treatment without cysteine. Exposure to 25 µM CTAB coating alone also significantly increased the dry weight content in comparison to control (Figure 5c). Seedlings treated with AgNO_3_ also exhibited increase in dry matter content compared to control; however, obtained values were significantly lower in comparison to AgNP-CTAB- exposures of corresponding concentrations. Addition of cysteine lowered the values only at the highest applied concentration of AgNO_3_ (Figure 5c). The weakest effect on dry matter content was observed after exposure to AgNP-PVP, after which the significant increase was recorded only after treatment with 100 µM AgNP-PVP (Figure 5c).

### 2.5. AgNP Uptake and Localisation

Silver accumulation in tobacco seedlings significantly grew with the increase of either AgNP or AgNO_3_ concentrations (Table 2). No significant difference in silver accumulation was observed between the corresponding concentrations of different treatments. Cysteine addition significantly reduced the silver uptake in all combined treatments (Table 2). This reduction was stronger after exposure to AgNO_3_ with cysteine compared to corresponding concentrations of combined treatments with either AgNP-PVP or AgNP-CTAB, although it was statistically significant only for the lowest applied concentration.

**Figure 6:**
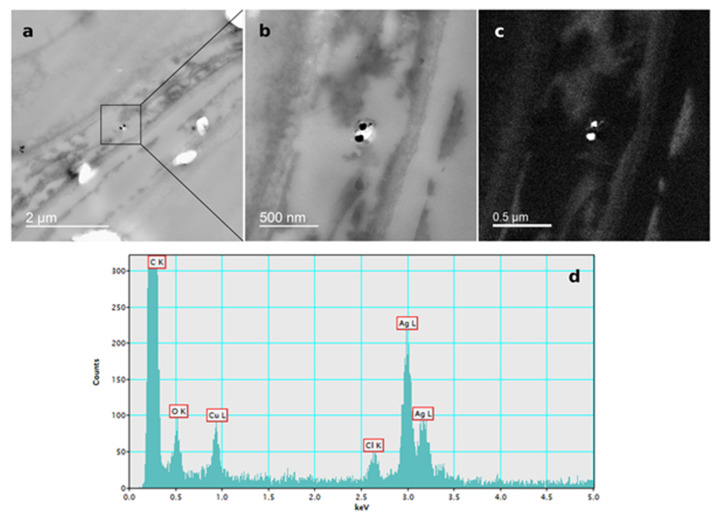
AgNP localization in the root cells of a three-week-old tobacco seedling after growing on a solid ½ strength MS medium supplemented with 100 μM AgNP-PVP. TEM images of (**a**) AgNPs in the epidermal root cell, (**b**) bright field image, (**c**) silver elemental map, (**d**) energy-dispersive X-ray spectrum.

**Figure 7:**
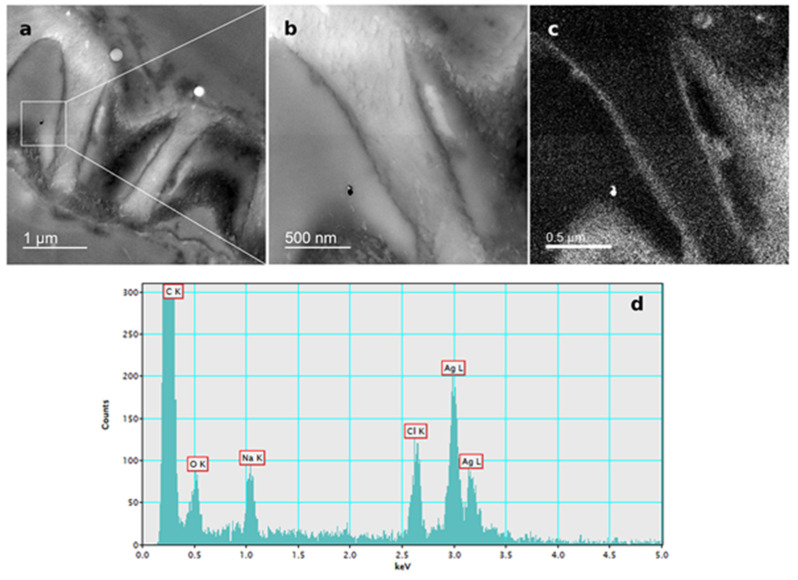
AgNP localization in the root cells of a three-week-old tobacco seedling after growing on a solid ½ strength MS medium supplemented with 100 μM AgNP-CTAB. TEM images of (**a**) AgNPs in the epidermal root cell, (**b**) bright field image, (**c**) silver elemental map, (**d**) energy-dispersive X-ray spectrum.

The presence of AgNPs in root cells of tobacco seedlings after exposure to 100 µM AgNP-PVP and AgNP-CTAB was confirmed by TEM-EDX analysis (Figure 6; Figure 7).

## 3. Discussion

Seed germination and early growth of seedlings are frequently used parameters to assess acute effects of stressful environmental conditions on plant physiology [29,30,31]. In the present study, these parameters were examined after exposure of tobacco seeds to AgNPs stabilised with two different coatings, PVP and CTAB as well as to AgNO_3_, applied in three different concentrations (25, 50 and 100 µM), which were chosen based on the results obtained in our previous studies. Namely, it was established that these concentrations of AgNPs and AgNO_3_ show different effects in induction of oxidative stress and activity of antioxidant enzymes in *Allium cepa* roots [17] as well as in tobacco seedlings [32] and adult plants [18]. Moreover, they were also found to result with different changes in organelles ultrastructure (particularly chloroplasts) and responses in up- and down-regulation of cell proteome, which were dependent on the applied concentration and the form of silver, but also on the plant developmental stage [19,32]. All abovementioned studies exhibited that the phytotoxicity of AgNPs differed from AgNO_3_-induced toxicity and was correlated with their surface coating and overall surface charge, but also with developmental stage of the certain plant species. Therefore, in this study we aimed to investigate the response of 25, 50, and 100 µM AgNP-PVP and AgNP-CTAB on germination and early growth of tobacco to obtain a better insight on how the same concentrations of differently coated AgNPs can affect the same plant species in different stages of its development. Obtained results revealed that CTAB-coated AgNPs had significant effect on all tested parameters, while AgNP-PVP significantly reduced root growth and fresh matter content. Negative effects of AgNPs on germination and plant early growth have been already reported by other authors; Stampoulis et al. [33] demonstrated inhibited seed germination and reduced biomass in zucchini, while Tripathi et al. [23] reported that germination, fresh and dry weight as well as root and shoot length of pea seedlings significantly declined after exposure to AgNPs. Similar results were also reported for broad bean [34], corn [22], wheat [35], rice [36], radish [37], pearl millet [21], barley [38], and mustard [39]. Furthermore, negative effects of AgNPs on germination were also found in cucumber and lettuce [40] as well as ryegrass, barley, and flax [41]. The mechanisms explaining AgNP-induced reduction in germination and plant biomass are still not elucidated; however, increased dry matter content induced by AgNP-CTAB in our study as well as decreased water content in AgNP-exposed radish seedlings [37], together with findings that AgNPs affect balance of water by changing the transcription of aquaporin genes [42], imply that AgNPs can affect plant growth by reducing the water uptake.

The silver uptake was similar at all corresponding concentrations of either AgNP-PVP or AgNP-CTAB and their uptake in root cell was confirmed by TEM-EDX, suggesting that toxic effects on tobacco seedlings were at least partially due to nanoparticulate form of AgNPs. However, effects observed after exposure to AgNP-PVP were not nearly as severe as those obtained in treatments with AgNP-CTAB; namely, AgNP-PVP had no effect on germination and exhibited a mild negative impact on root length and fresh weight. Discrepancy in the AgNP effects can be the consequence of the fact that impact of AgNPs on plants depends on various factors including species and growth stage as well as composition, concentration, and stability of AgNPs, etc. [24]. Yin et al. [43] suggested that the high toxicity of gum arabic-coated AgNPs compared to AgNO_3_ and particularly to AgNP-PVP on early growth of wetland plants was not only due to the presence of Ag^+^ ions, but it was more likely a result of the combination of size, coating and surface charge. In our study, the impact of AgNO_3_ on root growth was more pronounced compared to AgNP-PVP, but less severe than AgNP-CTAB even though the silver uptake was similar. Moreover, AgNO_3_ induced no significant changes in germination parameters of tobacco seeds and exhibited similar negative effects on fresh weight as corresponding AgNP-PVP-treatments. Furthermore, addition of cysteine, a strong silver ligand, successfully alleviated toxic effects of AgNO_3_ and AgNP-PVP-treatments on root growth, thus showing evidence that the root elongation induced by AgNP-PVP is mediated by Ag^+^ ions. Similar findings were reported in the study of Vannini et al. [28], in which cysteine completely rescued root elongation after rocket exposure to AgNP-PVP. Contrary, only a very weak alleviating effect was recorded after exposure to AgNP-CTAB, which confirms that released Ag^+^ ions have only a minor contribution to toxicity induced by CTAB-coated AgNPs. Stability analyses of AgNPs indicated some dissolution of both AgNP-PVP and AgNP-CTAB in a solid ½ strength MS medium. Releasing Ag^+^ ions and losing and/or displacing of the surface coating molecule are common changes that AgNPs can undergo in biological media [44]. Surface coating agents are non-covalently attached to AgNPs and are in an equilibrium state with the free ligand molecules in solution; dispersion of AgNPs in a biological medium can cause the surface coating agents to re-establish equilibrium by losing some of the coating molecules [45]. The addition of cysteine resulted in increased agglomeration of AgNPs and release of Ag^+^ ions, which was particularly pronounced in medium supplemented with AgNP-CTAB. Namely, CTAB is a relatively labile ligand in the presence of cysteine, with cysteine displacing CTAB through formation of a thiolate bond with surface silver atoms. Consequently, rapid agglomeration of AgNP-cysteine derives from a bridging effect through hydrogen boding between the amine and carboxylic acid groups of cysteine ligands on neighbouring nanoparticles [46]. A similar situation may arise in the case of cysteine added to AgNP-PVP, however the steric hindrance caused by the long chain polymers may significantly slow the rate at which bridging through hydrogen-bonding occurs and hence less agglomeration is noted. Further, silver clusters which may have formed can be relatively long lived in the presence of polyanions as clusters bind at anion groups within the polymer chains, concomitantly hindering those clusters from coming into contact [47]. In the present system, salts in ½ strength MS medium may mediate the formation of pseudo-polyanions from the interaction of PVP and cysteine which in turn stabilises small metal particles and clusters, hence another mechanism disfavouring agglomeration [48]. Moreover, the cysteine assay showed that Ag^+^ release was much faster from CTAB-coated AgNPs compared to AgNP-PVP, which may also indicate faster loss of surface coating in the case of AgNP-CTAB. Positively charged cetyltrimethylammonium cations released from AgNP-CTAB, accompanied with Ag^+^ ions, therefore could induce the severe toxic effects observed in this study and might explain why cysteine addition to treatments with AgNP-CTAB had even worse effects on root growth as well as fresh and dry weight compared to AgNP-CTAB alone. Furthermore, when solely CTAB was applied, a severe impact on most of the parameters was recorded, which indicates that the coating alone strongly contributes to AgNP-CTAB toxicity. Similarly, Wang et al. [49] found that gold nanorods, prepared using CTAB, were found to be highly toxic to human skin cells due to the presence of CTAB, not the gold nanorods themselves, with CTAB being toxic even at 10 µM. On the other hand, PVP alone exhibited either none or mild negative effects, which corroborates previous findings in *Eruca sativa* [28] and wheat [37]. However, there is also a possibility that observed toxicity of AgNPs is also correlated with their surface charge. Positively charged AgNPs, stabilised with branched polyethyleneimine (BPEI) or CTAB, were much more toxic for growth of bacteria compared to negatively charged AgNPs (citrate or SDS) or non-ionic (PVP) coatings [50,51], probably due to attachment of positively charged AgNPs to the negatively charged bacterial cell walls. Compared to AgNP-citrate and -PVP, BPEI-coated AgNPs were found to be more biocidal for both *Escherichia coli* and *Daphnia magna* due to their smaller particle size and greater charge differences between the AgNP surface and the biologic surface, thus leading to higher attractive forces and potentially promoting the toxicity [52]. Significantly higher toxicity of positively charged AgNPs was also reported for tobacco plants exposed to AgNP-CTAB compared to AgNP-PVP and AgNP-citrate [17] and wheat calli treated with AgNP-cystamine (carrying a positive charge) compared to AgNP-citrate [53]. Furthermore, the significantly higher toxicity of positively charged AgNPs compared to the also positively charged Ag^+^ ions from AgNO_3_ found in the study of El Badawy et al. [50] and in our study, supports the idea that physical interaction, which induces cellular membrane damage, could be the primary mechanism for AgNPs toxicity not just the chemical effect caused by Ag^+^ ions alone.

## 4. Materials and Methods

### 4.1. AgNPs Synthesis

For synthesis of silver nanoparticles coated with polyvinylpyrrolidone (AgNP-PVP), a silver nitrate-polymer solution was prepared by addition of 0.02 g AgNO_3_ (99.999% purity, Sigma-Aldrich, St. Louis, MO, USA) and 0.019 g PVP (average mol. wt. 40000 g·mol^−1^, Sigma-Aldrich) to 120 mL ultrapure water (ion-free Milli-Q water, 18.2 MΩ-cm resistivity, Merck Millipore, Billerica, MA, USA), and brought to boiling. A 5 mL aliquot of 1% aqueous sodium citrate (Sigma-Aldrich) solution was rapidly added and the solution brought back to boiling. After several minutes the solution changed colour from transparent to light yellow. The solution was cooled to room temperature and dialysed against ultrapure water in a 10 kDa molecular weight cut-off (MWCO) membrane tubing (Thermo Scientific, Waltham, MA, USA) for 24 h.

For synthesis of AgNPs coated with cetyltrimethylammonium bromide (AgNP-CTAB), silver nitrate-surfactant solution was prepared by addition of 0.02 g AgNO_3_ and 0.0043 g CTAB (Sigma Aldrich) to 62.5 mL ultrapure water. The AgNO_3_-CTAB solution was added to a solution of 0.01 g ascorbic acid (Sigma Aldrich) in 62.5 mL ultrapure water by burette in a constant stream under continuous stirring. Both solutions were pre-cooled to 0 °C prior to mixing. The combined solution changed colour from light yellow to orange with increasing addition of AgNO_3_. After addition of all AgNO_3_ solution, stirring was stopped and the nanoparticle dispersion was placed in a 10 kDa MWCO membrane tubing and dialysed against ultrapure water for 24 h.

### 4.2. Physico-Chemical Characterisation of AgNPs

Physico-chemical characterisation of AgNP-PVP and AgNP-CTAB stock solutions in ultrapure water was conducted as described in a previous study [29]. Briefly, formation of AgNPs was verified by the presence of a surface plasmon resonance (SPR) peak using a UV-Vis spectrophotometer (Unicam, Cheshire, UK). The hydrodynamic diameters of both types of AgNPs were measured using dynamic light scattering (DLS), while their ζ potential (charge) was evaluated by measuring electrophoretic light scattering (ELS) with a Zetasizer Nano ZS (Malvern Panalytical, Malvern, UK) equipped with a green laser (532 nm). Intensity of scattered light was detected at the angle of 173°. Zetasizer software 6.32 was used for data processing. The hydrodynamic diameters of AgNPs are given as the average value of 10 measurements (mean ± S.D., *n* = 10) and are reported as the volume size distributions; for the charge of AgNPs, results are reported as an average value of 5 measurements (mean ± S.D., *n* = 5).

The concentration of silver in both AgNP stock solutions (*n* = 5) was measured using ELAN DRC-e inductively coupled plasma mass spectrometer (ICP-MS) (Perkin Elmer, Waltham, MA, USA) before treatments to enable calculations for AgNP working solutions. Silver concentration was calculated according to the calibration curve obtained with a set of standards of known concentrations. Detection limits and limit of quantification (LOQ) were 0.2 and 1 mg·kg^−1^, respectively.

The possible silver dissolution from both AgNP-PVP and AgNP-CTAB in ultrapure water was determined by centrifugal ultrafiltration (Millipore Amicon Ultra-4 3K) through a membrane with a 3 kDa molecular weight limit. Suspensions were centrifuged for 30 min at 15,000× *g*. Total silver concentration in the AgNP suspension and the filtrates was determined in acidified solutions (10% *v*/*v* HNO_3_) using ELAN DRC-e ICP-MS. The silver concentration in the filtrate as determined by ICP-MS was related to the silver concentration before ultrafiltration to calculate dissolved Ag^+^ ions.

Imaging of the nanoparticles was carried out by pipetting 2 μL of each AgNP stock solution onto a Formvar^®^/Carbon copper grid, air-dried, and examined by monochromated TF20 (FEI Tecnai G2, FEI, Hillsboro, OR, USA) transmission electron microscope (TEM), with a Schottky cathode and operating at 200 kV. The TEM was equipped for energy-dispersive X-ray spectroscopy (EDX) with a SiLi detector and an ultrathin window. For each stock solution, four replicas (*n* = 4) were analysed.

### 4.3. Plant Material and Culture Treatments

This research was conducted in conditions of plant tissue culture using solidified ½ strength Murashige and Skoog (MS) nutrient medium (0.2% (*w*/*v*) Phytagel, 1.5% (*w*/*v*) sucrose; both Sigma Aldrich) [54]. Nutrient medium was sterilised and supplemented with AgNP-PVP, AgNP-CTAB or AgNO_3_, alone and in combination with cysteine (Sigma Aldrich; applied in a cysteine:silver molar ratio of 5), to obtain 25, 50, and 100 µM concentrations of silver and 125, 250, and 500 µM concentrations of cysteine, respectively. AgNO_3_ stock solution (100 mM) was prepared in ultrapure water. Additionally, media supplemented with 25 µM concentration of each coating (PVP or CTAB) were also tested. Prepared treatment solutions were quickly poured in Petri dishes (90 mm diameter) and left to solidify.

Tobacco (*Nicotiana tabacum* L.) cv. Burley seeds were surface sterilised for 15 min using 50% (*v*/*v*) NaOCl (Kemika), and then rinsed 3 times with sterile deionised H_2_O before being placed on the ½ strength solid MS medium. As a control, seeds were germinated on a ½ strength solid MS medium devoid of AgNPs, AgNO_3_, cysteine, or coatings. 100 seeds were sown on Petri dishes for each treatment. To promote and synchronise seed germination, Petri dishes were put on cold stratification (+4 °C) for 2 days before the beginning of the experiment [55]. During the experiment, all plant material was kept in the growth chamber at 24 ± 1 °C with 16/8 h light/dark cycles and 90 µmol m^−2^ s^−1^ light intensity. Germination was monitored for 5 consecutive days, starting from the 3rd day after seed stratification. After 3 weeks, seedlings were harvested and used for weight and root length measurements. All experiments were conducted three times.

### 4.4. AgNP Stability in Exposure Medium

Stability of 100 µM AgNPs (either AgNP-PVP or AgNP-CTAB) alone or in combination with 500 µM cysteine in the ½ strength solid MS medium with dissolved Phytagel was determined using UV-Vis spectrophotometry. Immediately after the medium was supplemented with AgNPs, with or without cysteine, a 1 mL aliquot of each solution was put in a 1 cm path-length quartz cuvette and left to solidify completely. Solidified ½ strength MS medium without AgNPs and cysteine was used as a reference. Spectrophotometric absorbance was measured in the range of 300–800 nm during the next 5 days, i.e., for the duration of germination experiment, and prepared cuvettes were stored under the same conditions as the plant material.

### 4.5. Seed Germination

Seed germination was monitored for five days, at the same time on each day. Seeds were considered germinated when the radicle emerged from the seed. In total 300 seeds (100 from each experiment) were analysed.

Germination percentage was calculated using the formula:

Germination percentage (%) = final number of germinated seeds/total number of seeds × 100(3)

Daily counts of germinated seeds were used to calculate germination index (GI) and germination rate (T_50_), using the formulas by Farooq et al. [56]:

GI = ∑ Nt (number of germinated seeds on the t day)/Dt (germination days),(4)

T_50_ = t_i_ + {[(N/2) − n_i_]*(t_i_ − t_j_)/n_i_ − n_j_}, (5)

where N is the final number of germinated seeds, n_i_ and n_j_ are cumulative number of seeds germinated by consecutive counts at times t_i_ and t_j_, considering that n_i_ < N/2 < n_j_. All germination parameters are presented as percentage compared to relevant control (non-treated sample).

### 4.6. Seedling Growth

For root length measurements, 60 randomly chosen seedlings from three experiments were analysed for each treatment (20 seedlings from each experiment). For measurements of fresh and dry weight, seedlings were pooled together to form 3 replicas per experiment due to low weight of 3-weeks old seedlings; in total there were 9 replicas for each treatment.

At the end of the experiment, seedlings were harvested, and the length of the main root was measured using a ruler. Subsequently, root length reduction percentage (RLPR) was calculated using the following formula [57]:

RLPR % = 100 × [1 − (root length _stress_/root length _control_)]. (6)

Fresh weight of seedlings was used to calculate the fresh weight reduction percentage (FWPR) [57] as follows:

FWPR % = 100 × [1 − (fresh weight _stress_/fresh weight _control_)]. (7)

Seedlings were oven dried at 60 °C for 24 h, and dry weight was used to determine dry matter content (DMC) percentage using the formula:

DMC % = 100 × [dry weight/(fresh weight + dry weight)].(8)

### 4.7. Silver Uptake and Localisation

Prior to sample preparation, 3-week old AgNP- and AgNO_3_-treated seedlings were washed with ultrapure water and subsequently oven-dried at 80 °C for 24 h to obtain a constant weight. Dried samples were prepared for analysis according to the modified US EPA method 3052 by microwave digestion; firstly in 10 mL concentrated HNO_3_ for 10 min at 130 °C, then for another 15 min at 180 °C. The next preparation step was digestion in 1 mL H_2_O_2_ for 5 min at 85 °C and subsequently for 4 min at 130 °C. Afterwards, the samples were cooled and diluted with 1% (*v*/*v*) HNO_3_ to obtain a total volume of 50 mL. Analyses were performed using ELAN DRC-e ICP-MS (Perkin Elmer, Waltham, MA, USA) to measure silver content. Silver concentration was as previously described in the previous section. Spike recovery tests were 93.5% for AgNO_3_-treated seedlings, and 91.8% and 94.5% for seedlings exposed to AgNP-PVP and AgNP-CTAB, respectively.

For localization of AgNPs in tobacco seedlings exposed to 100 μM concentrations of AgNP-PVP and AgNP-CTAB, tissue sections were prepared as previously reported [17,18]. Briefly, tissue was fixed with 1% (*w*/*v*) glutaraldehyde in 50 mM cacodylate buffer (pH 7.2) for 1 h at + 4 °C, washed twice with cold 50 mM cacodylate buffer (pH 7.2) and post-fixed with 1% (*w*/*v*) osmium tetroxide in the same buffer for 1 h at + 4 °C, followed by a 10-min wash in ice-cold water. Afterwards, tissue was dehydrated in a graded series of ethanol and subsequently embedded in Spurr’s resin. Ultrathin sections were stained with 2% (*w*/*v*) uranyl acetate and 2% (*w*/*v*) lead citrate and examined using monochromated TF20 (FEI Tecnai G2) TEM for confirmation of AgNPs uptake in the tobacco cells.

### 4.8. Statistical Analysis

Statistical analysis was performed using STATISTICA 13.0 (Stat Soft Inc., Palo Alto, CA, USA) software package. The data were analysed by either a two-way ANOVA to identify concentration and treatment effects or a one-way ANOVA to identify effects of cysteine addition and coatings alone. Differences between means were considered statistically significant at *p* ≤ 0.05 (Duncan post hoc test).

## 5. Conclusions

Coating-dependent effects of AgNP-PVP and AgNP-CTAB on tobacco germination and early growth were recorded. AgNP-PVP induced mild toxicity on early growth only at higher concentrations, while AgNP-CTAB caused severe negative, even compared to AgNO_3_. Cysteine addition successfully alleviated AgNP-PVP-induced negative effects, which suggests that the toxic impact of PVP-coated AgNPs is mainly due to release of Ag^+^ ions. On the contrary, cysteine failed to significantly improve germination and growth parameters after exposure to AgNP-CTAB. Since the CTAB coating alone had a negative effect on growth parameters, our results indicate that effects of CTAB-coated AgNPs can be ascribed to surface coating itself and that released Ag^+^ ions have only a minor contribution to its toxicity.

## Figures and Tables

**Table 1:** Physico-chemical characteristics of AgNP-PVP and AgNP-CTAB in ultrapure water by means of hydrodynamic diameter (d_H_) in nm obtained from size distribution by volume, ζ potential values in mV, surface plasmon resonance (SPR) peak in nm, and percentage of ionic Ag^+^.

Characteristics		AgNP-PVP	AgNP-CTAB
Size peak I	d_H_, nm	8.16 ± 0.37	26.28 ± 6.37
	Mean volume, %	81.28 ± 19.96	84.68 ± 12.49
Size peak II	d_H_, nm	41.53 ± 1.14	64.75 ± 7.28
	Mean volume, %	1.05 ± 0.96	6.10 ± 0.93
Size peak III	d_H_, nm	/	227.00 ± 16.02
	Mean volume, %	/	6.16 ± 3.52
ζ potential, mV		−1.0 ± 0.6	11.4 ± 1.1
SPR peak, nm		420	434
Ag^+^, %		0.3	0.5
Stocks concentrations, mM ^1^		9.8	11.5

^1^ Concentrations of both AgNP stock solutions were obtained by inductively coupled plasma mass spectrometer (ICP-MS).

**Table 2:** Silver content (µg g^−1^ dry weight (DW)) in tobacco seedlings after germination and 3-weeks growth on the ½ strength solid MS medium supplemented with 25, 50, and 100 μM AgNP-PVP, AgNP-CTAB, and AgNO_3_ and their combination with 125, 250, and 500 μM cysteine (cys).

		Ag content (µg g^−1^ DW)		
Treatment	Concentration (µM)	AgNP-PVP	AgNP-CTAB	AgNO_3_
Control	0	0 ^c1^	0 ^d^	0 ^d^
	25	46.71 ± 4.53 ^b,A^	45.18 ± 6.16 ^c,A^	42.89 ± 0.96 ^c,A^
Ag	50	71.86 ± 4.69 ^b,A^	73.39 ± 7.64 ^b,A^	66.72 ± 2.73 ^b,A^
	100	109.46 ± 16.00 ^a,A^	113.05 ± 6.93 ^a,A^	101.39 ± 3.82 ^a,A^
	25 + 125	31.18 ± 0.91 *^,#^	32.49 ± 4.55 ^#^	22.01 ± 1.02 *^,#,+^
Ag + cys	50 + 250	48.18 ± 0.68 *^,#^	49.20 ± 16.24 ^#^	29.20 ± 2.26 *^,#^
	100 + 500	89.43 ± 11.98 ^#^	75.00 ± 6.24 *^,#^	64.41 ± 5.73 *^,#^

^1^ Values are the means ± standard error of three biological replicates, each with three technical replicas. If columns are marked with different letters, the treatments are significantly different at *p* ≤ 0.05 (a two-way ANOVA followed by Duncan post-hoc test); small letters mark the differences among different concentrations of the same treatment type as well as control while capital letters mark the differences among different treatment types of the same concentration. Asterisk (*) denotes significant differences among treatments with and without cysteine of the corresponding concentration; hash sign (#) denotes significant difference between each treatment with cysteine and control, plus sign (+) denotes significant difference among treatments with cysteine of the corresponding concentration.

## Supplementary Materials

Supplementary materials can be found at https://www.mdpi.com/1422-0067/21/10/3441/s1. Figure S1: Effects of 25, 50 and 100 μM AgNP-PVP, AgNP-CTAB or AgNO_3_ and their combination with 125, 250 and 500 μM cysteine, and 25 μM PVP and CTAB coatings, added to a solid ½ strength MS medium, on germination and early growth of tobacco. Control, marked with red square, was common for all treatments.

## Abbreviations

AgNP	silver nanoparticles
PVP	polyvinylpyrrolidone
CTAB	cetyltrimethylammonium bromide
TEM	transmission electron microscopy
DLS	dynamic light scattering
ELS	electrophoretic light scattering
SPR	surface plasmon resonance
