# Supplementary figures and images for: Coating-Dependent Effects of Silver Nanoparticles on Tobacco Seed Germination and Early Growth
Source: Int J Mol Sci. 2020 May 13;21(10):3441. doi: 10.3390/ijms21103441 (PMC7279453; doi:10.3390/ijms21103441)

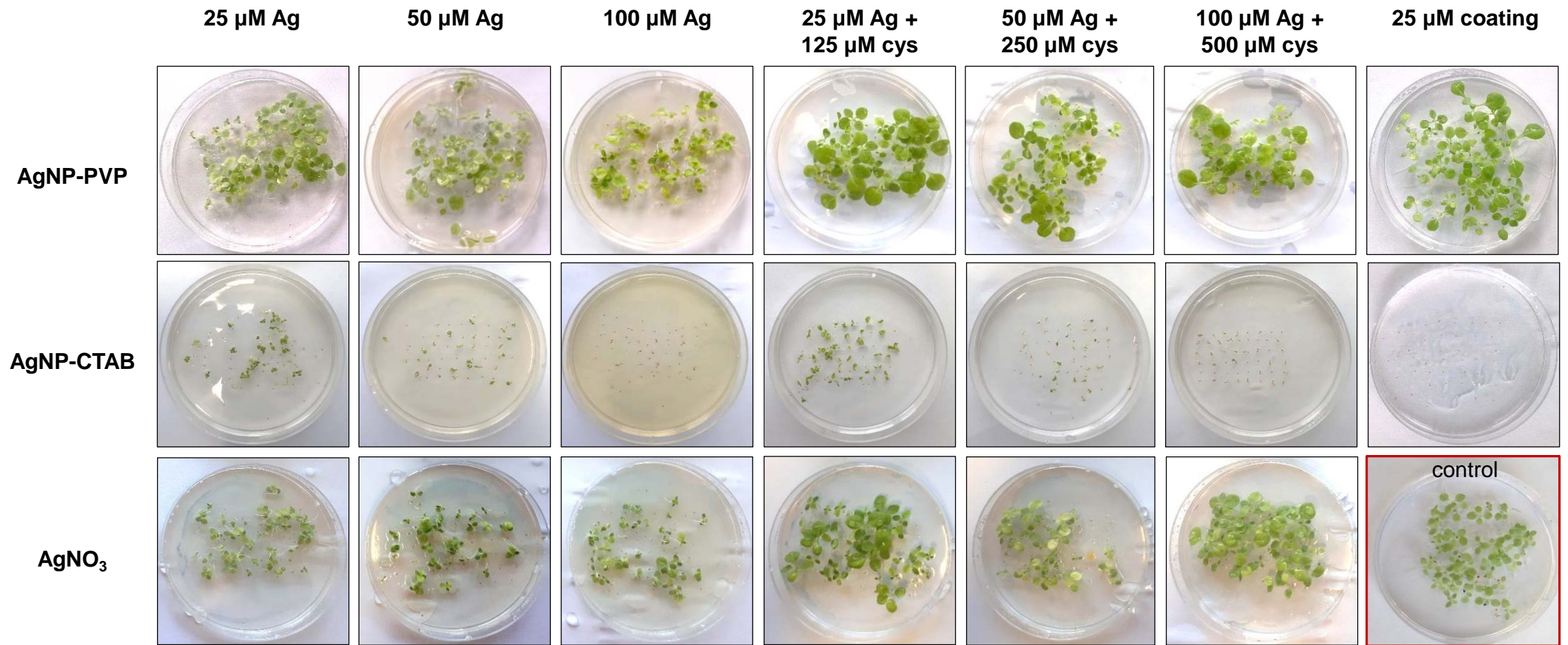

Supplement: Supplementary file 1 [file ijms-21-03441-s001.pdf]
